# Development and testing of the career decision-making self-efficacy scale for nursing students: a methodological study

**DOI:** 10.1186/s12912-022-01017-7

**Published:** 2022-08-23

**Authors:** Young-Mi Jung, In-Young Yoo

**Affiliations:** 1 Department of Nursing, Daegu Haany University, 1, Hanuidae-ro, Gyeongsan-si, Gyeongsangbuk-do 38610 Korea; 2 Department of Nursing, Jeonju University, 303, Cheonjam-ro, Wansan-gu, Jeollabuk-do 55069 Korea

**Keywords:** Career choice, Decision-making, Instrumentation, Self-efficacy, Nursing students

## Abstract

**Background:**

The conventional Career Decision-Making Self-Efficacy Scale does not reflect the situation in Korea due to different sociocultural attributes and fails to account for the unique nursing profession and changes in healthcare. We aimed to develop and psychometrically test the Career Decision-Making Self-Efficacy Scale for Nursing Students.

**Methods:**

A methodological study using a newly developed questionnaire tool and investigation of the validity and reliability of the preliminary instrument. Data were collected from 400 nursing students through an online survey conducted in May 2021. We identified 56 preliminary items through a literature review and focus group interviews. Of them, 40 were completed with a content validity index > .80. Content, construct, and criterion-related validity; internal consistency reliability; and test-retest reliability were used in the analysis.

**Results:**

Exploratory factor analysis revealed three factors including 21 items: adapting to work (20.5%), understanding the major (20.2%), and goal setting (16.4%), explaining 57.1% of the total variance. As a result of confirmatory factor analysis, 17 items in the three-factor structure were validated. Reliability, as verified by the test-retest interclass correlation coefficient, was .86 and Cronbach’s α was .92. The final Career Decision-Making Self-Efficacy Scale for Nursing Students consists of 17 items: adapting to work (7 items); understanding the major (4 items); and goal setting (6 items).

**Conclusion:**

The scale developed to measure the career decision-making self-efficacy of nursing students showed sufficient validity and reliability.

## Background

Career decision-making self-efficacy is the belief that one can successfully complete tasks necessary to make career decisions [1]. Self-efficacy refers to confidence regarding the ability to execute certain actions to attain a desired outcome, which affects achievement behavior and career decision-making [2]. Career decision-making self-efficacy plays a supporting role in exploring career paths, setting career goals, and making career decisions [3]. The average turnover rate among Korean nurses is 15%, which is higher than that of other occupations, and the turnover rate within 1 year among new graduate nurses is 42.7% [4]; similarly, the rate was 30.0–50.0% in the United States [5] and 8.1–27.8% in Taiwan [6]. Optimizing the structural work environment and cognitive aspects will improve the career identity of nurses by enhancing their career decision-making self-efficacy during their school years, increasing career adaptability, and leading to lower turnover [7].

Personal career development is explained by the social cognitive career theory, including the core concepts of self-efficacy, outcome expectations, and personal goals. The interaction between these variables affects personal career development [8]. A specific goal, based on the expectation of outcomes, is required to acquire career behavior. Identifying the influencing factors of career decision-making self-efficacy may indicate the time spent making a reasonable career decision and immersing in career behavior to achieve a goal [9].

The Career Decision-Making Self-Efficacy Scale (CDMSES) measures self-efficacy related to career decision-making tasks and behavior outcomes, as well as expectations for success, consisting of self-appraisal, occupational information, goal selection, planning, and problem solving [10]. The short form CDMSES (CDMSES-SF) consists of five items in five domains, for a total of 25 items, with items checked on a revised 5-level confidence continuum [11]. Validation studies of these tools showed sufficient internal reliability for all five domains [11]. However, only occupational information and goal selection were consistently identified in factor analysis [12, 13]. Moreover, a meta-analysis of these tools demonstrated stable reliability, but inconsistent sub-factors [13].

The CDMSES-SF validity and reliability were tested on Korean students, and the results were stable [14, 15]. It has also been used to measure career decision-making self-efficacy of Korean students [9, 16]. However, the CDMSES-SF in Korea was used without undergoing rigorous methodological procedures for tool development [9, 16]. Given that the CDMSES-SF was translated and surveyed for college students, it may not accurately examine the career efficacy of nursing students because questions about understanding the major or adapting to practice are not included among the sub-factors. Also, the testing of construct validity through discriminant validity assessment and confirmatory factor analysis was not presented in detail [14, 15]. Additionally, 20 years has passed since the validation of the CDMSES-SF. Due to different sociocultural attributes and failure to account for the uniqueness of the nursing profession and changes in healthcare, the conventional CDMSES cannot reflect the situation in Korea [17]. Students who enrolled in nursing majors without further considering their careers may have believed that nursing majors did not suit their aptitude, because of the burden and stress of nursing work [16]. Moreover, career decision-making self-efficacy is positively correlated with nursing students’ satisfaction with their major [18]. Thus, establishing measures to enhance satisfaction among nursing students by measuring their level of career decision-making self-efficacy and utilizing the findings during career counseling is required.

We aimed to develop a tool to measure career decision-making self-efficacy for nursing students. Our specific objectives were to 1) use qualitative research methods to identify initial items based on in-depth interviews; 2) conduct expert content validation on the initial items identified; 3) conduct a pilot study to construct the initial items; and 4) use quantitative research methods to apply the developed tool to nursing students and confirm the final items by testing the construct validity, criterion validity, and tool reliability.

## Methods

### Tool development

#### In-depth interviews for initial item identification

Interviews were conducted with ten fourth-year nursing students and three nurses with 3 years of clinical experience, who were conveniently sampled from July 14–23, 2020. Each participant was interviewed 1–2 times for approximately 60–120 minutes each. The main question was “Do you have the confidence or belief that you can be good at career decision-making and adaptation?” This study used the Colaizzi's phenomenological method, which is based on the fact that the participants' experiences cannot be directly observed, but can be described perceptually [19]. This approach ties common statements from participants and abstracts them step by step, focusing on deriving common characteristics of all research participants rather than individual properties. Therefore, Colaizzi's method was considered suitable to understand the essence of nursing students' career decisions and adaptation experiences. Answers were repeatedly read to extract meanings from statements, and items identified based on themes, theme clusters, and categories [19]. To ensure validity and trustworthiness, interview data were analyzed according to the evaluative criteria for rigor (truth value, applicability, consistency, and neutrality) proposed by Lincoln et al. (1985) [20]. The results were divided into five categories: 1) Career preparation activities: self-understanding, job values and preferences, and experience; 2) Career exploration: major exploration, difficulties in adapting to major, and collection of various information; 3) Career decision-making: stress coping ability, clinical practice adaptation ability, and employment barriers; 4) Career planning: career goal setting, planning, and concerns; and 5) Career certainty: confidence and uncertainty about career decision-making.

#### Content validity testing

In the first expert content validity testing, three items were deleted because of duplicate meanings from 56 preliminary items. The second expert content validity testing was conducted by 10 participants (six nursing professors and four nurse practitioners). The tool for measuring the content validity index (CVI) included 53 items: 10, career preparation activities; 15, career exploration; 17, career decision-making; 6, career planning; and 5, career certainty. The expert panel graded each item on a 4-point Likert scale. To determine the level of agreement among experts, item-level CVI (I-CVI) and the percentage of responses with 3 or 4 points for each item, with an I-CVI ≥.78 indicating good content validity, were calculated [21]. All items exceeded the cut-off value and I-CVI values ranged between .90 and 1.00 to satisfy the criterion. For scale-level CVI (S-CVI), S-CVI divided by average variance extracted (AVE) was 0.98, satisfying the cut-off of ≥.90. However, the expert opinion gathering process identified ambiguous or duplicated items, and items requiring changed sub-factors. Consequently, the tool was revised to 40 items (career preparation activities contained 12 items; 10, career exploration; 10, career decision-making; 4 career planning; 4, career certainty). To ensure the accuracy of vocabulary and appropriateness of expressions, word order or postpositions were revised based on advice from one Korean language professor. Um and Cho (2005) reported that a distribution error may occur when respondents with a neutral opinion about an item were forced to choose a positive or negative response [22]. Therefore, we used a 5-point Likert scale with an option to choose “average” as the response. Higher scores indicated a higher level of career decision-making self-efficacy.

#### Pilot study

A pilot study was conducted on 39 nursing students with an average age of 21.28±.75 years. The items identified included 40 items (career preparation activities contained 12 items; 10, career exploration; 9, career decision-making; 4, career planning; 5, career certainty). Reliability was calculated using Cronbach’s alpha (α). The preliminary tool’s overall reliability was α=.95, while each domain’s reliability was .85, .89, .78, .78, and .91 for career preparation activities, career exploration, career decision-making, career planning, and career certainty, respectively.

### Tool assessment

#### Study population

The target population for this study was students in Korea enrolled in nursing major, while the accessible population was nursing students enrolled in nursing education institutions in “D” or “J” city. The sample size required should be at least 5–10 times the number of scale items to test the validity of the tool based on exploratory factor analysis (EFA) and confirmatory factor analysis (CFA) [23]. The Career Decision-Making Self-Efficacy Scale for Nursing students (CDMSES-NS) contained 40 items, thus requiring a minimum of 200–400 participants. Considering dropouts and incomplete responses, the target study population was set to 400 participants maximally. The inclusion criteria were being able to communicate, being alert, understanding the purpose and content of the study, and agreeing to participate voluntarily. Participants were recruited through an online questionnaire survey, and no dropouts or incomplete responses occurred.

#### Data collection

The data collection period was May 13–31, 2021. Participants were recruited through announcements posted in Internet cafés used by nursing students from two private colleges. A link to the CDMSES (https://forms.gle/EN5rFdhPhb5XFVbi6) was attached to the recruitment announcement.

#### Measurement tools

To test convergent validity, we used the Career Decision Scale originally developed by Osipow et al. (1976), adapted to Korean by Kho (1993) [24, 25]. It consists of 18 items graded on a 5-point Likert scale and scored in reverse, with higher scores allowing decision making regarding career choice easy. Its reliability at the time of development was .90 and .82 for test-retest correlation on two different groups of college students, respectively [24], while the reliability was α=.86.

To test convergent validity, we also used the Vocational Identity Scale originally developed by Holland et al. (1980) for the My Vocational Situation, adapted to Korean by Kim and Kim (1997) [26, 27]. It consists of 18 items graded on a 4-point Likert scale, with higher scores indicating a stronger career identity. Its reliability at the time of development was .89 for the test-retest correlation for two different groups of high school students [26], while the reliability was α=.91.

Furthermore, to test convergent validity, we used the Career Preparation Behavior Scale developed by Kim and Kim (1997) for college students, consisting of 16 items graded on a 4-point Likert scale, with higher scores indicating more active career preparation behavior [27]. Its reliability at the time of development was α=.84 [27], while the reliability was α=.90.

To test concurrent validity, we used the CDMSES-SF, consisting of 25 items in four domains (goal selection, occupational information, future planning, problem solving) with higher scores indicating higher levels of career decision-making self-efficacy. Its reliability at the time of development was α=.93 and the reliability of the domains was α=.69–.83 [1]. In our study, the reliability of the CDMSES-SF was α=.92, and the reliability of the domains was α=.74–.88.

To test discriminant validity, we used the Korean Career Barrier Inventory originally developed by Kim (2002) to measure career barriers [28]. The tool consists of 25 items graded on a 4-point Likert scale, with higher scores indicating greater career barriers. Its reliability at the time of development was α=.85 [28], and in this study was α=.92.

#### Data analysis

Data analysis was performed using SPSS/WIN version 26.0 (SPSS Inc., Chicago, USA) and Mplus 7.4 programs (Muthén & Muthén, Los Angeles, CA, USA). The demographic characteristics of the participants were analyzed using descriptive statistics. In the item analysis for the developed tool, the degree of bias was checked using normality testing based on the assessment of the mean, standard deviation (SD), skewness, and kurtosis of each item. EFA including Kaiser-Meyer-Olkin (KMO), and Bartlett’s sphericity and CFA were performed to test the construct validity, and convergent and discriminant validity were identified. In CFA, the fitness index was assessed using chi-square (χ^2^), normed χ^2^ (chi-square minimum/degree of freedom [CMIN/DF]), comparative fit index (CFI), goodness of fit index (GFI), Tucker-Lewis Index (TLI), root mean square error of approximation (RMSEA), and standardized root mean residual (SRMR). To test the convergent, discriminant, and concurrent validity, the correlations between the scores measured by the CDMSES-NS and the scores measured by tools for career decision-making, career identity, career preparation behavior, career decision-making self-efficacy, and career barriers were assessed using Pearson’s correlation coefficients. To test the stability reliability, the intraclass correlation coefficient (ICC), representing test-retest reliability, was measured and assessed. To test the homogeneity reliability, the corrected item total correlation (ITC) and internal consistency reliability (Cronbach’s α coefficient) were assessed.

### Ethical considerations

The methods were approved by the Jeonju University Institution Review Board (IRB No. jjIRB-210409-HR-2021-0408). All procedures performed in studies involving human participants were in accordance with the ethical standards of the Declaration of Helsinki. Informed consent was obtained from all individual participants involved in the study. Before starting the online questionnaire survey, all participants were informed about the objectives and methods of the study, the right to withdraw participation from the study, and use and confidentiality of the collected data.

## Results

### General characteristics

The study included 82.2% women and 17.8% men. First, second, third, and fourth-year college students constituted 25.0%, 28.4%, 23.3%, and 23.3%, respectively, of the participants (Table 1). Regarding religion, “None” was the most common response (71.2%), and 54.5% responded “Yes” to extracurricular activities. Among students, 72% expressed the desire to work as a clinical nurse after graduation.

**Table 1:** Demographic characteristics of participants (*N*=400)

Characteristics	Categories	Frequency	Percentage
Gender	Male	71	17.8
	Female	329	82.2
College level	1^st^ year	100	25.0
	2^nd^ year	114	28.4
	3^rd^ year	93	23.3
	4^th^ year	93	23.3
Religion	Protestant	59	14.8
	Catholic	21	5.2
	Buddhist	31	7.8
	None	285	71.2
	Other	4	1.0
Extracurricular activities	Yes	218	54.5
	No	182	45.5
Hope to work as a clinical nurse after graduation	Yes	288	72.0
	No	112	28.0
Total		400	100.0

### Item analysis

Each item’s mean score range was 3.03–4.22 points (SD, 0.68–1.10), skewness range was -1.04–0.02, while the kurtosis range was -0.79–2.42. These values satisfy the criteria for normality as they were below the absolute values of 3 and 10, respectively [29].

### Validity analysis

#### Construct validity

The KMO was .92, indicating the presence of common latent factors between the items. Bartlett’s sphericity test results showed χ^2^=7560.59 (*p*<.001), indicating that the items were significantly correlated and suitable for factor analysis. Of 40 items with common variance ≤.40, 15 were excluded from the analysis. The appropriate number of factors was determined by considering the scree graphs, total explained variance of ≥50%, and reliability of the constructed items. When the number of factors was set to four, those loaded in the first, second, third, and fourth factor were seven, seven, seven, and four, respectively. The total explained variance was 58.3%, and two items in the fourth factor showed a factor loading value of <.50. When the number of factors was set to three, four items with common variance ≤.40 were removed. Subsequently, the number of items extracted from the first, second, and third factors was seven, eight, and six, respectively. The total explained variance was 57.1%, and all 21 items showed a factor loading value of ≥.50. Accordingly, the number of factors was set to three. When the EFA suitability was checked, the KMO measure was .92, and Bartlett’s sphericity test result was χ^2^=4564.74 (*p*<.001), confirming that the correlation matrix between the items was not an identity matrix, and that the items in the tool were suitable for factor analysis. The explained variances of factors 1, 2, and 3 were 20.5%, 20.2%, and 16.4%, respectively, indicating an even distribution (<40.0%) without being sed toward one factor (Table 2). CFA was performed to test the construct validity of the three factors and 21 items identified by the EFA, by testing the relationships between the items and latent variables. After excluding four additional items with factor loadings of <.50 or those that caused a drop in the AVE index and construct validity [4, 16, 19, 20], 17 items were selected (Table 3). When the fitness of the model was analyzed with the final 17 items, the results showed χ^2^=194.86 (*p*<.001), CMIN/DF=2.14, CFI=.97, GFI=.91, TLI=.96, RMSEA=.05, and SRMR=.04, indicating that all indices satisfied the criteria, excluding the χ^2^ index, which is sensitive to the sample size [29]. The validity of the measurement tool was verified by construct reliability (CR) and AVE. The CR values’ range was .81–.88, exceeding the cut-off value of .70, indicating internal consistency of measured variables for latent variables. The AVE of latent variables’ range was .50–.53. The AVE value of latent variables was higher than the square of the correlation between constructs, indicating discriminant validity for the constructs. The AVE values (.50–.53) were higher than the coefficient of determination of the three latent variables (.40–.47), confirming the presence of discriminant validity. Sub-factors were renamed based on the common characteristics of the items grouped by factor. Factor 1, “adapting to work”, consisted of seven items to identify adaptation to clinical practice or confidence in work performance in the nursing profession. Factor 2, “understanding the major”, consisted of four items to identify understanding the characteristics of nursing, confidence about completing the curriculum, and the core competencies that nurses should have. Factor 3, “goal setting”, consisted of six items to identify career goals, career planning, and employment preparation. The scores measured by the developed tool showed a strong positive correlation with career decision-making (*r*=.53, *p*<.001) and career identity (*r*=.53, *p*<.001), moderately positive correlation with career preparation behavior (*r*=.36, *p*<.001), and strong negative correlation with career barriers (*r*=-.64, *p*<.001). Accordingly, the convergent and discriminant validity were verified.

**Table 2:** Exploratory factor analysis of career efficacy scale for Korean nursing students (*N*=400)

Item	Factors		
	1	2	3
38. I can adapt well to my workplace after being hired	.82	.15	.24
39. I have confidence in performing nursing work	.78	.16	.31
40. I can adapt well even if actual nursing work is different than what I had thought	.72	.12	.20
36. I am satisfied with my career choice	.68	.34	-.09
37. I have a positive conviction about my career decision-making	.67	.41	.04
30. I can adapt well to clinical practice	.65	.27	.24
31. I can cope with problems that occur in nursing practice	.58	.15	.44
14. I can successfully complete nursing theory curriculum	.22	.74	.13
15. I can successfully complete nursing practical training	.37	.69	.11
16. Nursing major courses have been helpful in my career adaptation (school life, employment, etc.)	.29	.68	.05
13. I have a good understanding of the characteristics of nursing (e.g., theoretical and practical training, extracurricular activities)	.22	.68	.20
19. I can collect information through a variety of sources (e.g., acquaintances, the internet)	-.01	.60	.35
4. I actively participate in extracurricular activities	.22	.60	.11
20. I am capable of collecting specific information about nursing career that suits my aptitude	.02	.53	.49
12. I have the core competencies that a nurse should have (e.g., knowledge integration application, communication and collaboration, critical thinking skills, leadership, etc.).	.37	.53	.32
9. I am good at writing a personal essay	.12	-.01	.75
10. I am good at job interviews	.21	.05	.71
34. I can set my own career roadmap	.09	.39	.63
11. I can prepare well for the career that I choose	.40	.32	.60
32. I can set my own career goals	.25	.39	.56
33. I can make my own career plans	.19	.42	.56
Eigen value	4.30	4.24	3.45
Explained variance (%)	20.5	20.2	16.4
Total explained variance (%)	20.5	40.7	57.1

**Table 3:** Confirmatory factor analysis of career efficacy scale for Korean nursing students (*N*=400)

Item	Factors	Standardized estimates		SE	C.R.	p	CR	AVE
30	Adapting to work	.68		.03	22.36	<.001	.88	.52
31		.67		.03	22.06	<.001		
36		.61		.04	17.26	<.001		
37		.67		.03	21.26	<.001		
38		.83		.02	43.52	<.001		
39		.84		.02	44.16	<.001		
40		.72		.03	25.72	<.001		
12	Understanding the major	.76		.03	25.21	<.001	.82	.53
13		.74		.03	24.54	<.001		
14		.68		.04	17.62	<.001		
15		.73		.03	22.99	<.001		
9	Goal setting	.50		.05	9.41	<.001	.86	.51
10		.56		.05	11.00	<.001		
11		.81		.04	20.06	<.001		
32		.84		.04	22.31	<.001		
33		.81		.04	20.60	<.001		
34		.67		.04	18.24	<.001		
Fitness index	χ^2^ (p)	CMIN/DF	CFI	GFI	TLI	RMSEA		SRMR
Criteria	(> .05)	≤3	≥.90	≥.90	≥.90	≤.10		≤.08
Model	194.86 (*p*<.001)	2.14	.97	.91	.96	.05		.04

*AVE* average variance extracted, *CFI* comparative fit index, *CMIN/DF* Chi-square minimum/degree of freedom, *C.R.* critical ratio, *CR* construct reliability, *GFI* goodness of fit index, *RMSEA* root mean square error of approximation, *SE* standard error, *SRMR* standardized root mean residual, *TLI* Tucker-Lewis index

#### Concurrent validity

When the correlation between the CDMSES-SF and the tool developed in this study was analyzed for concurrent validity, the results showed a strong positive correlation (*r*=.66, *p*<.001).

### Reliability analysis

#### Stability reliability

To test the tool stability, a retest was conducted on the same 40 nursing students participating in the main questionnaire survey, using the same tool two weeks after the initial survey. The reliability between the two sets of measures, calculated using ICC, was .87 (95% CI: 0.75–0.93), .85 (95% CI: 0.72–0.92), and .69 (95% CI: 0.41–0.83) for adapting to work, understanding the major, and goal setting, respectively. The reliability of the entire tool was .86 (95% CI: 0.73–0.93), indicating very high stability reliability (Table 4).

**Table 4:** Reliability for career efficacy scale for Korean nursing students (*N*=400)

Factors	Test-retest (*N*=40)					
	Test score (M±SD)	Retest score (M±SD)	PCC r (p)	ICC (95% CI)	M±SD	Cronbach’s α
Adapting to work	3.60±.76	3.83±.67	.77 (<.001)	.87 (.75–.93)	3.74±.63	.90
Understanding the major	3.91±.57	3.96±.67	.75 (<.001)	.85 (.72–.92)	3.89±.61	.90
Goal setting	3.59±.68	3.78±.60	.53 (<.001)	.69 (.41–.83)	3.56±.59	.86
Total	3.67±.59	3.85±.56	.75 (<.001)	.86 (.73–.93)	3.71±.52	.92

*CI* confidence interval, *ICC* intraclass correlation coefficient, *M* mean, *PCC* Pearson correlation coefficient, *SD* standard deviation

#### Homogeneity reliability

Internal consistency was assessed using the ITC and Cronbach’s α coefficient. The range of ITC values constructed by each factor was .66–.85 (Table 4). The values were ≥|.30|; thus, satisfying the criteria [30]. Meanwhile, positive correlations were found between all the items constructed by each factor. Cronbach’s α of all 17 items inputted in the analysis was .92, while that of sub-factors adapting to work, understanding the major, and goal setting were .90, .90, and .86, respectively.

## Discussion

In the EFA to test the construct validity of the developed tool, the total explained variance was 57.1%, while that of factors 1, 2, and 3 was evenly distributed (20.5%, 20.2%, and 16.4%, respectively). Our results confirmed that the three sub-factors adequately explained career decision-making self-efficacy. In contrast, the 50-item CDMSES widely used in Korea had a total explained variance of 52.0% at the time of development, while the variance rate of the five sub-factors was 16.9% (self-appraisal), 11.4% (occupational information), 10.7% (goal selection), 8.1% (plans for the future), and 4.9% (problem solving) [10]. In the CDMSES-SF, occupational information and goal selection factors strongly appeared [1], while future planning was included in each factor of problem solving. Furthermore, self-assessment factors were distributed in duplicate regarding occupational information and goal selection factors. In a study validating the CDMSES-SF in Korean [15], 25 items were extracted from four sub-factors of goal selection, occupational information, problem solving, and future planning. Therefore, even if the items are similar, conceptual factors may be extracted differently in environments with different sociocultural backgrounds. The items excluded in the EFA had a common variance of <.40 and belonged to cross-factor loadings. These items did not represent each subfactor, and the constructs were mixed. Consequently, they were excluded.

Career decision-making self-efficacy among nursing students identified by factor analysis consisted of “adapting to work,” “understanding the major,” and “goal setting,” with each showing similar explanatory power. For the factor “adapting to work,” the CDMSES did not include items related to confidence in clinical practice and work performance, because it was developed based on foreign college students, while studies validating the CDMSES for Korean middle and high school and college students identified constructs such as those found in the CDMSES [14, 15]. Our findings do not confirm these results. Factors related to adapting to work showed the highest variance ratio, demonstrating the uniqueness and importance of nursing as a practical discipline and profession. Adapting to work in the nursing profession is markedly affecting job satisfaction and role transition for students and new graduates [16, 31]. Nursing students experience stress from excessive academic workload and clinical practice [32, 33]. Moreover, new graduates also show difficulties in adapting to clinical settings, experiencing reality shock because of the gap between the knowledge or skills learned in school and nursing needs and various roles expected in clinical settings [31]. Therefore, assessment of adapting to work among nursing students or new graduate nurses may be an indicator for identifying the achievement behaviors or outcomes regarding their careers.

For “understanding the major,” the CDMSES did not have separate factors about the major, but the level of understanding the major was measured using items such as “I can find out what the required curriculum is for completing my major.” Low career identity and preparation behavior during nursing student years lead to low job satisfaction and high turnover rates among new graduate nurses [34]. Moreover, because of the expanded role of nurses, including infectious disease management since the COVID-19 pandemic, there is a growing need to develop nursing skills to address diverse roles [35]. To achieve this, the educational goals set in nursing education programs should be linked to modern nursing competency levels, while core nursing competencies should be set based on the goals and learnability of nursing education programs and composition of curriculum [36]. Therefore, understanding the major is interpreted as a key factor in explaining the uniqueness of nursing.

In “goal setting,” everyone needs to set specific goals to carry out career behavior. Such goals should be based on outcome expectations obtained through expectations and behaviors about self-efficacy [27]. Our results confirm those of previous studies [1, 14, 15, 37], reporting that goal planning was identified as a construct for measuring career decision-making self-efficacy. Everyone has a different purpose, attitude, and motivation in the career process and lead their lives according to set goals. A career may be a subject and situation constructed according to individual aims [38]. Career decision-making self-efficacy varies significantly according to the level of goal orientation that one should achieve [39].

The CDMSES-NS we developed showed a strong positive correlation with career decision-making and career identity, and moderately positive correlation with career preparation behavior used to test convergent validity. Our results confirmed a study reporting a correlation coefficient between CDMSES and career identity [40]. Moreover, the correlation coefficient was higher than that in a previous study [41], when career decision was reverse-coded for measuring career indecision. Higher correlation between variables is determined to show higher convergent validity [29]. Therefore, the convergent validity of the tool we found was assured. Conversely, our tool showed a lower correlation coefficient with career preparation behavior than that previously reported [42]. Therefore, thoroughly investigating the validity between career preparation and the CDMSES-NS we developed is required. Meanwhile, the analysis of discriminant validity showed a higher correlation coefficient than that previously reported [43]. Accordingly, the discriminant validity of the tool we developed was confirmed.

The analysis of concurrent validity among criterion validity showed a strong positive correlation between the CDMSES-NS and CDMSES-SF. Regarding each factor, only “understanding the major” showed a moderately positive correlation, while “adapting to work” and “goal setting” showed strong positive correlations. Therefore, “adapting to work” and “understanding the major” are key factors in measuring career decision-making self-efficacy among nursing students and may be differentiated to measure career decision-making self-efficacy among regular college students. We verified only concurrent validity among criterion validity, but future studies should also test predictive validity using data regarding job satisfaction, nursing professionalism, and turnover rate among nurses. The analysis of stability reliability by test-retest of the tool we used showed very high reliability, while the CDMSES-NS satisfied the criteria for stability and homogeneity of reliability. Korean adolescents lack confidence in their decision-making regarding career choices, and this characteristic persists across college students [14]. Nursing students with a fixed career path after graduation may not have the opportunity to seek an insightful career education on various nursing tasks and be informed of their broader role in community and health care institutions as well as clinical settings [18]. With nurses registering for clinical work after graduation at an exceedingly higher turnover rate than other professionals, it is necessary to establish nursing professionalism and career identity for long-term career development of nursing students [44]. Therefore, the CDMSES-NS, which can measure nursing students’ career development and career competency, can provide basic data to understand nursing students’ career attitude tendencies that influence their career success.

This study had two limitations. First, it is difficult to generalize the study results because the tool development and sampling for the questionnaire survey targeted students from private nursing colleges. Second, our results are hardly generalizable to different academic majors outside nursing. Third, there may be limitations and risks of bias inherent in the study design. However, the tool we used was developed with consideration for the cultural characteristics and environment of Korean nursing students and has research significance for use in measuring career development and enhancing career decision-making self-efficacy among Korean nursing students.

## Conclusions

The CDMSES-NS we developed was tested for validity and reliability using various methods. Based on our findings, we provide several suggestions. First, repeat studies are warranted to assess the tool validity and reliability through large-scale cohort surveys on nursing students in education institutions by type (public versus private) and region (capital region versus non-capital region), as well as on college students with majors other than nursing. Furthermore, follow-up studies using the tool are recommended to test the effects of nursing career education studies or intervention programs related to career decision-making self-efficacy among nursing students. Moreover, psychometric tests with the simultaneous application of the tool should be performed to measure career decision-making self-efficacy among college students in Korea and abroad.

## Data Availability

The datasets generated and/or analysed during the current study are not publicly available due to the sensitive nature of the questions asked in this study. Additionally, the survey respondents were assured raw data would remain confidential and would not be shared. The data is available from the corresponding author on reasonable request.

## Abbreviations

CDMSES: Career Decision-Making Self-Efficacy Scale
CDMSES-SF: short form CDMSES
CVI: Content validity index
I-CVI: Item-level CVI
S-CVI: Scale-level CVI
AVE: Average variance extracted
EFA: Exploratory factor analysis
CDMSES-NS: Career Decision-Making Self-Efficacy Scale for Nursing students
SD: Standard deviation
KMO: Kaiser-Meyer-Olkin
CFA: Confirmatory factor analysis
CFI: Comparative fit index
CMIN/DF: chi-square minimum/degree of freedom
GFI: Goodness of fit index
TLI: Tucker-Lewis Index
RMSEA: Root mean square error of approximation
SRMR: Standardized root mean residual
ICC: Intraclass correlation coefficient
ITC: Corrected item total correlation
C.R.: Critical ratio
CR: Construct reliability
CI: Confidence interval
M: Mean
PCC: Pearson correlation coefficient
